# Lack of association between angiotensin-converting enzyme inhibitors and angiotensin receptor blockers and pain improvement in patients with oral cancer

**DOI:** 10.3332/ecancer.2020.1121

**Published:** 2020-10-13

**Authors:** Kim N Du, Andrew J Shepherd, Irvin V Ma, Carlos J Roldan, Moran Amit, Lei M S Feng, Shubh Desai, Juan P Cata

**Affiliations:** 1 Department of Internal Medicine, Baylor College of Medicine – Houston, Texas 77030, USA; 2 Department of Symptom Research, The University of Texas MD Anderson Cancer Center – Houston, Texas 77030, USA; 3 University of Nevada, Reno School of Medicine, Las Vegas, NV 89557, USA; 4 Department of Pain Medicine, The University of Texas MD Anderson Cancer Center – Houston, Texas 77030, USA; 5 Department of Head and Neck Surgery, The University of Texas MD Anderson Cancer Center – Houston, Texas 77030, USA; 6 Department of Biostatistics, The University of Texas MD Anderson Cancer Center – Houston, Texas 77030, USA; 7 Department of Anesthesiology and Pain Medicine, The University of Texas MD Anderson Cancer Center – Houston, Texas 77030, USA; 8 Anesthesiology and Surgical Oncology Research Group, Houston, Texas 77401, USA

**Keywords:** oral cancer, pain, angiotensin receptor blockers

## Abstract

**Background:**

There is a growing body of literature implicating angiotensin II in the modulation of tumour-associated inflammation and pain. However, the impact of angiotensin-converting enzyme inhibitors (ACEis) and angiotensin II receptor blockers (ARBs) on pain and inflammation has not yet been studied in oral cancers. The objective is to investigate the role of ACEi and ARB pharmacotherapy on preoperative pain and inflammatory biomarkers, neutrophil-to-lymphocyte ratio (NLR), platelet-to-lymphocyte ratio (PLR) and monocyte-to-lymphocyte ratio (MLR), in patients with oral cancer.

**Methods:**

We performed a retrospective study on patients who underwent oral cancer surgery. The Wilcoxon rank-sum test or Kruskal–Wallis analysis was used to evaluate differences in demographic, tumour-related and preoperative characteristics and amongst patients using ARBs, ACEis and no treatment. Multivariable analysis was fitted to estimate the effects of important covariates on severe preoperative pain.

**Results:**

A total of 162 patients with oral malignancies were included in the study. After adjusting for significant covariates, patients with perineural invasion were found to have higher levels of pain (*p* = 0.0278). Similarly, patients taking ARBs were found to have lower levels of perineural invasion (*p* = 0.035). The analysis did not demonstrate a significant difference in pain levels when comparing ARBs or ACEis to the no treatment group (*p* = 0.250). Furthermore, the use of ARB or ACEi did not significantly alter preoperative NLR (*p* = 0.701) or MLR (*p* = 0.869).

**Conclusions:**

When compared to no treatment, ARBs and ACEis are not associated with significant analgesic effect or decreased inflammatory scores (NLR, PLR and MLR).

## Introduction

Oral and lip cancers rank amongst the 15th most common malignancies worldwide [1]. Approximately 550,050 patients in the world are diagnosed with oral cancers each year [1]. Oral cancers are more common amongst men, older age groups and those with cofactors such as tobacco and alcohol use [2]. This patient population is also at risk for cardiovascular comorbidities. Therefore, angiotensin II receptor blockers (ARBs, i.e., losartan, valsartan and omelsartan) and angiotensin-converting enzyme inhibitors (ACEis, i.e., enalapril and linsinopril) are commonly prescribed to patients with oral cancers to treat hypertension and heart failure or prevent hypertensive nephropathy [3].

Previous studies indicate that ACEis and ARBs modulate important signalling mechanisms involved in inflammatory and neuropathic pain [4–7]. Pain is one of the most common complaints of patients with oral malignancies and can be present even before any cancer treatment has been initiated [8]. The severity of pain in patients with oral cancer is rated as more intense than pain produced by other malignancies as it interferes with essential body functions including eating, talking and swallowing, and can be refractory to conventional treatments [9].

Multiple mechanisms appear to mediate pain in oral cancer including inflammation and nerve invasion. Preclinical studies by Scheff *et al* [10] indicate that tumour necrosis factor-alpha (TNF-α), a known inflammatory cytokine, significantly contributes to tongue allodynia in mice injected with supernatant media from oral cancer cells. TNF-α also upregulates chemokine ligand 2 (CCL2), a monocyte chemoattractant implicated in the potentiation of perineural invasion [11, 12]. Bakst *et al* [12] demonstrated that CCL2 secreted by the nerves recruits inflammatory monocytes to differentiate into macrophages and propagates perineural invasion in mouse cancer models.

In addition, other nociceptive mediators such as nerve growth factor (NGF) also promote nociception in mice bearing oral cancers. Ye *et al* [13] demonstrated in mouse models that NGF is implicated in changes in the expression of the transient vanilloid receptor 1 (TRPV1) in trigeminal ganglion cells, suggesting an interaction between NGF and TRPV1. The expression of NGF in the neurons is regulated by the angiotensin II (AT2) receptor which, in turn, can modulate the function of TRPV1 [6]. Moreover, the antagonism of the AT2 receptor also decreases the infiltration of CD3+ T cells and macrophages in the dorsal root ganglion, which correlates with a reduction in allodynia in animals with neuropathic pain [6, 14, 15]. Therefore, it appears that the AT2/NGF/TRPV1 interaction is an important mechanism in mediating nociception and inflammation. Currently, the possible impact of ACEis and ARBs drugs on pain associated with oral cancer has not been established.

The study was designed to evaluate a possible modulation role of ARBs and ACEis in pain associated with oral cancers. Due to the neuropathic and the inflammatory components of pain associated with this pathology, we hypothesised that patients actively taking ARBs and ACEis would present with lower pain intensity before surgery. Second, we also investigated whether the use of ARBs or ACEis had any impact on inflammatory markers. Since AT2 is a known inflammatory mediator via CD3+ T cells and macrophages, we hypothesised that patients taking ARBs and ACEis would present with lower levels of inflammation and decreased leucocyte proliferation, which we assessed via the neutrophil-to-lymphocyte ratio (NLR), platelet-to-lymphocyte ratio (PLR) and monocyte-to-lymphocyte ratio (MLR).

## Methods

After approval from the University of Texas MD Anderson Cancer Centre Institutional Review Board (#PA16-1033), we performed a retrospective study that included a cohort of patients with oral cancers who underwent surgical treatment between January 2004 and January 2018. Patients of 18 years of age or older were included, whereas patients with positive HPV status and those with missing information regarding demographics, tumour pathology, preoperative pain, preoperative pharmacotherapy and living status were excluded.

Information collected from the electronic medical records included age, gender, body mass index (BMI), the American Society of Anaesthesiologist’s (ASA) physical classification, stage of disease, history of smoking, tumour location, presence of perineural invasion, neoadjuvant chemotherapy, preoperative analgesics, preoperative analgesic use (any) and preoperative blood pressure pharmacotherapy (ACEi, ARBs and no treatment). Recorded dependent variables included self-reported preoperative orofacial pain intensity using a verbal numeric rating scale (VNRS; 0–3 = mild pain, 4–6 = moderate pain and 7–10 = severe pain) at the time of anaesthesia assessment, preoperative NLR, PLR and preoperative MLR.

### Outcomes

On analysing the effects of ARBs and ACEis on inflammation and nociception, the primary outcomes of this study were (1) preoperative oral cancer pain scores as rated by the VNRS and (2) level of inflammation as indicated by biomarkers, NLR, PLR and MLR. Preoperative NLR was defined as the ratio between the absolute neutrophil count and absolute lymphocyte count of routine blood samples drawn within 14 days prior to the date of surgery, closest to time of surgery. Preoperative MLR was defined as the ratio between the absolute monocyte count and absolute lymphocyte count of blood samples drawn within 14 days prior to the date of surgery, closest to time of surgery. The same strategy was applied to calculate NLR and PLR.

### Statistical analysis

Summary statistics including mean, standard deviation, median and range for continuous variables such as age, BMI, preoperative haemoglobin, NLR, PLR and MLR and frequency counts and percentages for categorical variables such as ASA, T stage, N stage, severe preoperative oral cancer pain and recurrence/death status are provided. The Chi-square test or Fisher’s exact test was used to evaluate the association between two categorical variables. The Wilcoxon rank-sum test or Kruskal–Wallis test was used to evaluate the difference in a continuous variable between/amongst patient groups. A multivariable logistic regression model was fitted to estimate the effects of important covariates on severe preoperative pain. A *p*-value < 0.05 was considered to be statistically significant. The statistical software SAS 9.4 (SAS, Cary, NC) and S-Plus 8.2 (TIBCO Software Inc., Palo Alto, CA) were used for all the analyses.

## Results

**Figure 1.**
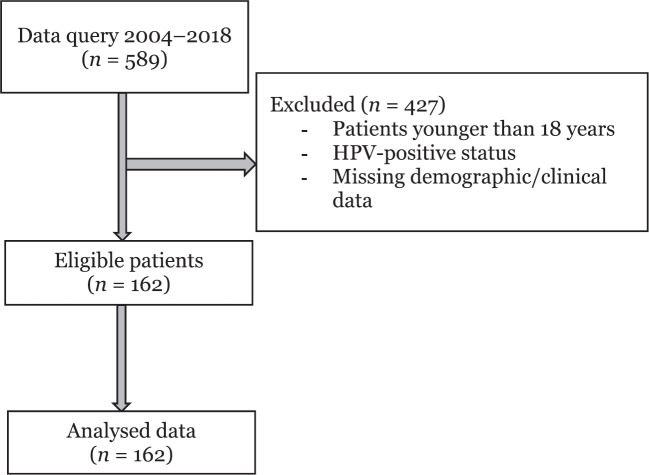
Consort flow diagram.

Of the 589 patients who underwent surgical resection for oral cancer, 162 patients met inclusion criteria (Figure 1). The demographic and clinical characteristics of patients based on their use of ARBs, ACEis or neither pharmacotherapy (none) are shown in Table 1. The median age was 60 years (range: 18–92), and the median BMI was 26.2 (range: 14.1–46.3). There were 124 males, and the majority had a history of cigarette smoking (97.1%) and an ASA physical status of 3 (85.1%) (Table 1). The most common primary tumour location was the tongue (56.5%). Other oral cancer locations included the floor of mouth (11.8%), alveolar ridge (9.9%), retromandibular trigone (8.7%), buccal mucosa (7.5%), hard palate (1.9%), gingiva (1.2%) and lip mucosa (1.2%). In this cohort, 72.8% of the patients (*n* = 118) were not actively taking ARB or ACEi pharmacotherapy. Amongst the others, 20.4% were taking ACEis (*n* = 33), and 6.8% of patients were taking ARBs (*n* = 11).

From the Kruskal–Wallis test or Fisher’s exact test, we observed statistically significant differences in age, tumour location and perineural invasion amongst treatment groups (Table 1). Patients taking ARBs had the highest median age when compared to the other two groups (65 versus 62 for ACEi and 58 for none; *p* = 0.015). The most common ARBs used were losartan (72.7%), followed by valsartan (18.18%) and irbesartan (9.09%). Amongst patients taking ACEIs, lisinopril was the most frequently used (84.8%) followed by enalapril (9.09%) and benazepril (6.06%). Interestingly, patients taking ARBs also had the lowest rate of perineural invasion (9.1% versus 30.3% for ACEi and 44.1% for none; *p* = 0.035), as well as the lowest percentage of tumoural location at the tongue (27.3% versus 39.4% for ACEi and 64.1% for none; *p* = 0.006). We did not detect a significant difference in the rate of neoadjuvant chemotherapy, analgesics or non-steroidal analgesic drug (NSAID) use amongst different treatment groups.

### Oral cancer pain by pharmacotherapies (ACEis, ARBs or none)

In this cohort, the majority of patients (73.1%, *n* = 117) experienced mild levels of preoperative pain (VNRS: 0–3), whereas 16.3% of patients (*n* = 26) reported moderate levels of preoperative pain (VNRS: 4–6), and only 10.6% (*n* = 17) reported severe pain (VNRS ≥ 7). More than half (*n* = 93, 57.4%) of the patients were taking analgesics at the time of surgery. The rate of moderate-to-severe preoperative pain was higher for patients with stage N1–N4 tumours compared to patients presenting in stage N0 (34.1% versus 18.7%; *p* = 0.028). Furthermore, the univariate analysis indicated that individuals with tumours with perineural invasion more commonly reported severe pain (*p* = 0.017). Patients taking ARBs were found to have the lowest level of perineural invasion (90.0% versus 69.7% in ACEi and 55.9% in none, *p* = 0.351) (Table 1).

The analysis did not demonstrate a statistically significant change in pain intensity when comparing the use of ARBs or ACEis to the no treatment group (*p* = 0.251) (Table 2). After excluding patients using analgesics preoperatively (*n* = 90), the analysis indicated that the proportion of subjects reporting moderate-to-severe pain was higher in the ARB (37%) group than in the two other cohorts of patients (ACEIs = 3% and none = 2%, *p* = 0.004). Due to the small sample of patients reporting severe pain (VNRS ≥ 7; *n* = 17), the multivariable logistic regression model was limited to two covariates. With the adjustment of tumour location, the odds of severe preoperative pain for patients with perineural invasion was 3.938 times higher (OR = 3.938, 95% CI for OR: 1.335, 11.615; *p*-value = 0.013) than patients without perineural invasion (Table 3).

### Inflammatory biomarkers (NLR and MLR) by pharmacotherapies (ACEis, ARBs or none)

On analysing the effects of ARBs and ACEis on the inflammatory pathway, we utilised biomarkers, NLR, PLR and MLR, to indicate levels of leucocyte proliferation. As shown in Table 2, the Kruskal–Wallis test did not indicate a significant difference in levels of preoperative biomarkers such as NLR (*p* = 0.701), PLR (*p* = 0.482) or MLR (*p* = 0.869) amongst treatment groups, even after excluding analgesic use. Similarly, the preoperative NLR (*p* = 0.231), PLR (*p* = 0.852) and MLR (*p* = 0.933) were not found to be significantly different amongst patients with severe preoperative pain when compared to patients with mild-to-moderate preoperative pain. Interestingly, patients taking ARBs had the highest mean preoperative NLR (3.187) and preoperative MLR (0.529) when compared to the ACEi and no treatment group, but this was not observed for PLR values (Table 2).

## Discussion

Pain remains an unresolved health problem in patients with oral cancer. This study suggests that ACEis and ARBs are not associated with significant analgesic effects when compared to no treatment. Interestingly, the rate of patients reporting moderate-to-severe pain, after excluding those taking analgesics from the whole cohort, was higher in the ARB group. This finding could suggest that the use of ARBs is associated with higher pain; however, the analysis was limited by the small sample size.

The association between the use of ARBs and functional outcomes has been investigated in other human pain conditions including total knee arthroplasty, migraine and chemotherapy-induced neuropathy [16–18]. Patients using ARBs did not show any improvement in knee flexion scores after total knee arthroplasty and volunteers with experimental ischaemic pain [4, 16]. However, in other pathologies such as migraine, Diener *et al* [17] demonstrated that telmisartan reduced pain severity compared to placebo. Interestingly, the use of ACEis and ARBs was associated with some protection of myelinated fibre function in a mixed population of patients with chemotherapy-induced neuropathy [18].

There are several potential explanations for the findings. First, Scheff *et al* [10] reported that inflammation is a main driver of the mechanism of pain in oral cancers. In support of that notion, a retrospective study demonstrated that non-steroidal anti-inflammatory drugs showed some analgesic effects in patients with oral cancer [19]. The modulatory effects of ARBs on the inflammatory response are well documented; however, their anti-inflammatory activity appears to be drug specific (telmisartan > irbesartan > valsartan and losartan) [20, 21]. In this study, we investigated whether ARBs or ACEis had any impact on inflammatory scores. Interestingly, we found no difference in those markers of inflammation. We can speculate that the results are confounded by the fact that we grouped several ARBs in the analysis; thus, we were not able to detect a significant effect on inflammatory scores.

Second, the selectivity of ARBs against the angiotensin II type I receptors (AT2R1) and angiotensin II type II receptors (AT2R2) should be considered. With the exception of losartan, all ARBs are highly selective for the AT2R1 and show 10,000–30,000 times greater affinity for this receptor than for AT2R2. As a result of ARBs’ high selectivity, the AT2R2 can be exposed to a higher concentration of ATII counteracting the effects of AT2R1 [22]. In addition, previous preclinical studies have indicated that the proinflammatory effects of the renin–angiotensin system result from AT2R1 stimulation at the target organ, and thus, it is plausible that systemic blockade of AT2R1 signalling has opposing effects on different tissues [23].

Third, patients in this cohort taking ARBs were less likely to have perineural invasion, and tumours without perineural invasion were less likely to report severe pain. However, we did not find a significant reduction in severe pain amongst patients taking ARBs. It is important to consider that perineural invasion is a complex process highly regulated by multiple nociceptive mediators which are predominantly inflammatory cytokines, chemokines and growth factors [24]. Although perineural invasion encompasses both inflammatory and neuropathic insults, oral cancer ARBs appear to be more effective in the modulation of neuropathic than inflammatory pain which may explain the lack of significant effect in pain improvement [22]. Shepherd *et al* [15] demonstrated that ARBs ameliorated pain behaviours in animals with spared nerve injury rather than those with inflammatory pain. In that study, the authors suggested that AT2R signalling on peripheral macrophages was an indispensable drive for the development of chronic neuropathic pain but not inflammatory pain [15]. Other investigations support Shepherd’s findings. For instance, ARBs showed anti-allodynic effects in animals with vincristine- and paclitaxel-induced neuropathic pain [7, 25]. In our cohort of patients, the level of bioinflammatory markers, NLR and MLR did not differ significantly amongst ACEi and ARB treatment groups when compared to no treatment, again supporting the lack of efficacy of ARBs on inflammatory pain even after excluding patients taking NSAIDs.

Finally, previous data suggest that angiotensin-converting enzyme inhibitors inhibit the enzyme dipeptidyl carboxypeptidase, thus blocking the degradation of nociceptive kinins such as bradykinin and substance P [26, 27]. In animals with paclitaxel-induced neuropathy, enalapril worsened neuropathic pain by increasing the concentrations of bradykinin-related peptides in the sciatic nerve [28]. Contrarily, ramipril decreased the pain perception thresholds to ischaemic pain in humans and hypertensive rats as well as in those with neuropathic pain after constriction nerve injury, suggesting a potential analgesic effect of ACEis.[4, 5, 29] However, these medications did not have any impact on acute postsurgical pain in a large cohort of patients [30].

This work has several limitations including the retrospective design and the small number of patients receiving ARBs and ACEis which may have affected the statistical analysis. Furthermore, due to the small number of patients receiving those medications, dose-response studies or any investigation to underpin their effect on the primary outcome was not possible. Another limitation of this study is that we did not account for differences in baseline levels of plasma AT2 between treatment and control groups. AT2 is known to be elevated in patients with hypertension [31]. However, the patients in our control group consisted of both hypertensive and normotensive patients. Thus, it may be plausible that the elevated AT2 levels in hypertensive patients taking ACEis and ARBs were simply reduced to normotensive levels, negating any differences in potential anti-inflammatory or analgesic effects between treatment and control groups.

## Conclusion

In conclusion, this study suggests that the use of ACEis or ARBs is not associated with a significant effect on pain in patients with oral cancers. However, patients taking ARBs were shown to have lower levels of perineural invasion. Additional studies should be performed to establish whether ARB-induced reduction in perineural invasion is due to direct effect on cancer cells or via an indirect effect on infiltration leucocytes.

## Conflicts of interest

The author(s) declare that they have no conflicts of interest.

## Funding statement

This study was supported by internal departmental funds and NIH P30CA016672 (MD Anderson Cancer Center).

## Figures and Tables

**Table 1.** Demographic characteristics and tumour-related variables.

Variable		ACEIs	ARBs	None	*p*-value
Age, mean (SD)		63.09 (11.95)	66.9 (7.93)	57.35 (14.87)	0.014
Gender	Female	6 (18.2)	1 (9.1)	31 (26.3)	0.359
	Male	27 (81.8)	10 (90.9)	87 (73.7)	
Body mass index, *n* (%)	≤25	9 (27.3)	4 (36.4)	50 (42.7)	0.273
	>25	24 (72.7)	7 (63.6)	67 (57.3)	
ASA, *n* (%)	2	2 (6.1)	0 (0)	20 (17.1)	0.132
	3/4	31 (93.9)	11 (100)	97 (82.9)	
History of smoking, *n* (%)	Current	11 (45.8)	4 (66.7)	51 (68)	0.162
	Past history	11 (45.8)	2 (33.3)	23 (30.7)	
	None	2 (8.3)	0 (0)	1 (1.3)	
Tumour location, *n* (%)	Tongue Other	13 (39.4) 20 (60.6)	3 (27.3) 8 (72.7)	75 (64.1) 42 (35.9)	0.006
Tumour staging, *n* (%)	1/2	16 (48.5)	6 (54.5)	43 (36.4)	0.291
	3/4	17 (51.5)	5 (45.5)	75 (63.6)	
Node staging, *n* (%)	N0	14 (42.4)	5 (45.5)	57 (48.3)	0.828
	N1–N4	19 (57.6)	6 (54.5)	61 (51.7)	
Perineural invasion, *n* (%)	No	23 (69.7)	10 (90.9)	66 (55.9)	0.035
	Yes	10 (30.3)	1 (9.1)	52 (44.1)	
Neoadjuvant	No	28 (84.8)	10 (90.9)	92 (98)	0.445
chemotherapy, *n* (%)	Yes	5 (15.2)	1 (9.1)	26 (22)	
Any analgesic, *n* (%)	No	15 (45.5)	6 (54.5)	46 (39)	0.542
	Yes	18 (54.5)	5 (45.5)	72 (61)	
NSAIDs, *n* (%)	No	31 (93.9)	10 (90.9)	105 (89.9)	0.693
	Yes	2 (6.1)	1 (9.1)	13 (11.1)	

ACEIs: Angiotensin-converting enzyme inhibitors; ARBs: Angiotensin receptor blockers; ASA: American Society of Anaesthesiologists; NSAIDs: Non-steroidal anti-inflammatory drugs

**Table 2.** Effect of ACEIs and ARBs on preoperative pain and inflammatory scores.

Variable	ACEIs Mean (SD)	ARBs Mean (SD)	None Mean (SD)	*p*-value
Pain VNRS	1.66 (2.94)	2.72 (2.61)	1.91 (2.71)	0.251
NLR all patients Non-NSAID patients	2.79 (1.61) 2.84 (1.37)	3.18 (2.24) 2.52 (1.37)	2.94 (2.58) 2.56 (0.89)	0.701 0.901
MLR all patients Non-NSAID patients	0.4 (0.19) 0.37 (0.15)	0.52 (0.67) 0.31 (0.09)	0.39 (0.22) 0.36 (0.14)	0.869 0.871
PLR all patients Non-NSAID patients	134.8 (71.1) 143.1 (79.5)	130 (52.3) 137.8 (57.8)	156.1 (99.86) 135.4 (56.58)	0.482 0.97

VNRS: verbal numeric rating scale; NLR: neutrophil-to-lymphocyte ratio; MLR: Monocyte-to-lymphocyte ratio; PLR: platelet-to-lymphocyte ratio; ACEIs: Angiotensin-converting enzyme inhibitors; ARBs: Angiotensin receptor blockers; NSAIDs: Non-steroidal analgesic drugs

**Table 3.** Multivariable analysis of factors associated with severe pain.

Odds ratio estimates and Wald confidence intervals				
Effect	*p*-Value	OR	95% CI for OR	
Tumour location: other versus tongue	0.0364	3.182	1.076	9.414
Perineural invasion: yes versus no	0.0130	3.938	1.335	11.615

OR: Odds ratio; CI: Confidence interval
